# Co-infection With Hepatitis B in Tuberculosis Patients on Anti-tuberculosis Treatment and the Final Outcome

**DOI:** 10.7759/cureus.14433

**Published:** 2021-04-12

**Authors:** Amir F Khan, Ahsan Sajjad, Dedaar A Mian, Muhammad M Tariq, Usman K Jadoon, Muhammad Abbas, Kinza Shakeel, Nadia Saeed, Kiran Abbas

**Affiliations:** 1 Surgery, Abbas Institute of Medical Sciences, Muzaffarabad, PAK; 2 Surgery, Khyber Teaching Hospital, Peshawar, PAK; 3 Medicine, Lady Reading Hospital Medical Teaching Institute (MTI), Peshawar, PAK; 4 Surgery, Hayatabad Medical Complex, Peshawar, PAK; 5 Community Medicine, Khyber Medical College, Peshawar, PAK; 6 Surgery, Lady Reading Hospital, Peshawar, PAK; 7 Zoology, Women University of Azad Jammu & Kashmir, Azad Jammu & Kashmir, PAK; 8 Medicine, Jinnah Postgraduate Medical Centre, Karachi, PAK

**Keywords:** tuberculosis, hepatitis b virus, drug-induced liver injury, liver failure, clinical outcome

## Abstract

Background

The occurrence of both tuberculosis (TB) and concomitant hepatitis B virus (HBV) is likely to be associated with poor patient outcomes and poor treatment response.

Objective

To assess whether tuberculosis patients with concomitant hepatitis B virus infection were prone to poorer outcomes and treatment response.

Methodology

A case-control study was undertaken at the Tuberculosis Centre, DHQ Bagh Azad Kashmir and Pulmonology Department, Lady Reading Hospital, Peshawar, between March 2020 and August 2020. All patients with diagnosed tuberculosis and coinfection with hepatitis B were labeled as the case group while those with only tuberculosis acted as the control. All patients with tuberculosis were managed on a directly observed treatment strategy (DOTS). Non-compliant patients and those without complete data were excluded from the study. All data regarding socio-demographics, laboratory investigations, and clinical characteristics were recorded in a predefined proforma. Patients were considered to have good treatment outcomes when patients completed the treatment or had a negative smear at six months of treatment. The Statistical Package for the Social Sciences (SPSS) version 26 (IBM Corp, Armonk, NY) was used for the data analysis.

Results

A total of 178 patients were enrolled in the study. It was found that patients with concomitant hepatitis B had significantly poorer outcomes as compared to patients who did not have hepatitis B (<0.001). Similarly, TB and hepatitis B patients were significantly associated with severe tuberculosis (<0.001) and required a higher frequency of retreatment (<0.001).

Conclusion

Our study reports a strong association between the treatment response of patients with tuberculosis with an added hepatitis B infection. Furthermore, a larger number of patients with hepatitis B had severe tuberculosis as compared to those without hepatitis B.

## Introduction

Hepatitis B infection is a liver disease that causes inflammation and destruction of hepatocytes. Hepatitis B is responsible for 250 million infections globally and killed approximately 800,000 patients in 2015 [1-2]. Tuberculosis (TB) is another major global health concern causing 1.6 million deaths each year and 10 million new cases per year [3].

Chronic hepatitis B virus (HBV)﻿ infections in patients with tuberculosis range from 0.5% to 44% [4-5]. The occurrence of both tuberculosis and concomitant hepatitis B virus is associated with poor patient outcomes and poor treatment response. The incidence of the concomitant disease is not known in Pakistan; however, both diseases have a high incidence rate in Pakistan [6-7]. Due to the fact that chronic hepatitis B causes liver damage, the treatment for TB﻿ patients coinfected with hepatitis B virus remains a challenge in Pakistan since hepatotoxicity with antituberculous drugs is common [8-9].

Presently, isoniazid, rifampin, pyrazinamide, and ethambutol are considered the first-line drugs. These drugs are associated with hepatotoxicity﻿ exclusive to ethambutol. The occurrence of hepatotoxicity and other adverse effects ranges between 3% and 28% [10]. TB patients with hepatitis B infection are at higher risk of hepatotoxicity [9-10]. There are only limited data available on the effects of anti-tuberculous treatment in patients with coinfection with hepatitis B from the local setting. Therefore, the current study aimed at evaluating the treatment response and clinical outcome in TB patients with HBV coinfection.

## Materials and methods

A case-control study was undertaken at the Tuberculosis Centre, DHQ Bagh Azad Kashmir and Pulmonology Department, Lady Reading Hospital, Peshawar, between March 2020 and August 2020. The study was started after ethical approval was obtained from the institutional review board (reference number F-NU/IRB/3254675). A non-probability consecutive sampling technique was used to select participants for the study. All patients with diagnosed tuberculosis (TB) were eligible to participate after informed verbal consent was acquired. Patients with multiple drug-resistant (MDR) strains of tuberculosis were excluded from the study. All patients with tuberculosis were managed on a directly observed treatment strategy (DOTS) and were administered a combined pharmacotherapy of isoniazid (INH), rifampicin (RMP), pyrazinamide (PZA), with ethambutol as an add-on drug for the first two months of therapy followed by a dual drug combination of INH and RMP for the next four to six months. The severity of tuberculosis was based on sputum, chest X-ray, and chest cavity scores [11]. Grade 1 tuberculosis was defined as mild while both Grades 2 and 3 were labeled as severe TB. Patients were followed for six months. Patients with acute, acute resolving, or recovered infection of hepatitis B were excluded from the study. Noncompliant patients and those without complete data were not included in the final analysis. All data regarding socio-demographics, laboratory investigations, and clinical characteristics were recorded in a predefined proforma. All baseline tests were performed prior to the initiation of therapy. Workup for hepatitis B virus included hepatitis B surface antigen (HBsAg), total anti-hepatitis B core (HBC), immunoglobulin M (IgM), anti-HBC, anti-hepatitis B surface (HBS), and HBV deoxyribonucleic acid (DNA). Patients with chronic HBV infection were included in the study. An equal number of TB patients without HBV infection were enrolled to act as the control group. Patients were assessed for poor outcomes and treatment responses on a regular basis and at the end of the sixth month of treatment. A sorbent assay (enzyme-linked immunoassay (ELIZA)) test was performed to screen the presence of hepatitis B. A standard protocol was applied. All patients who completed the six months course and were negative for tuberculosis were considered to have a good treatment outcome. A bad treatment outcome was considered when the treatment had to be stopped due to intolerance to medications or if patients had a positive smear at the end of the six-month anti-tuberculosis treatment (ATT) course.

The Statistical Package for the Social Sciences (SPSS) version 26 (IBM Corp, Armonk, NY) was used for data analysis. All continuous data were presented using mean and standard deviation, whereas all categorical data were presented as frequency and percentages. A chi-square test was used to compare the outcome and treatment response between those with concomitant hepatitis B and those without hepatitis B. A p-value of < 0.05 was considered statistically significant.

## Results

A total of 178 patients were enrolled in the study. Eighty-nine (89) patients were hepatitis B positive and 89 were hepatitis B negative. The majority of the patients were between the ages of 20 and 34 years. The majority of the patients with TB and concomitant hepatitis B belonged to the rural area (63; 70.79%) (Table 1).

**Table 1:** Demographics and clinical characteristics of the study population (n=178)

Variables	Hepatitis B Positive	Hepatitis B Negative	Total
Sex			
Female	45 (50.56%)	48 (53.93%)	93 (52.25%)
Male	44 (49.44%)	41 (46.07%)	85 (47.75%)
Age			
20-34	22 (24.72%)	25 (28.09%)	47 (26.40%)
35-49	48 (53.93%)	43 (48.31%)	91 (51.12%)
≥50	19 (21.35%)	21 (23.60%)	40 (22.47%)
Tobacco user			
No	46 (51.69%)	53 (59.55%)	99 (55.62%)
Yes	43 (48.31%)	36 (40.45%)	79 (44.38%)
Resident			
Rural	63 (70.79%)	59 (66.29%)	122 (68.54%)
Urban	26 (29.21%)	30 (33.71%)	56 (31.46%)
Education			
Illiterate	9 (10.11%)	7 (7.87%)	16 (8.99%)
Under Secondary	72 (80.90%)	73 (82.02%)	145 (81.46%)
Above Secondary Education	8 (8.99%)	9 (10.11%)	17 (9.55%)

**Figure 1:**
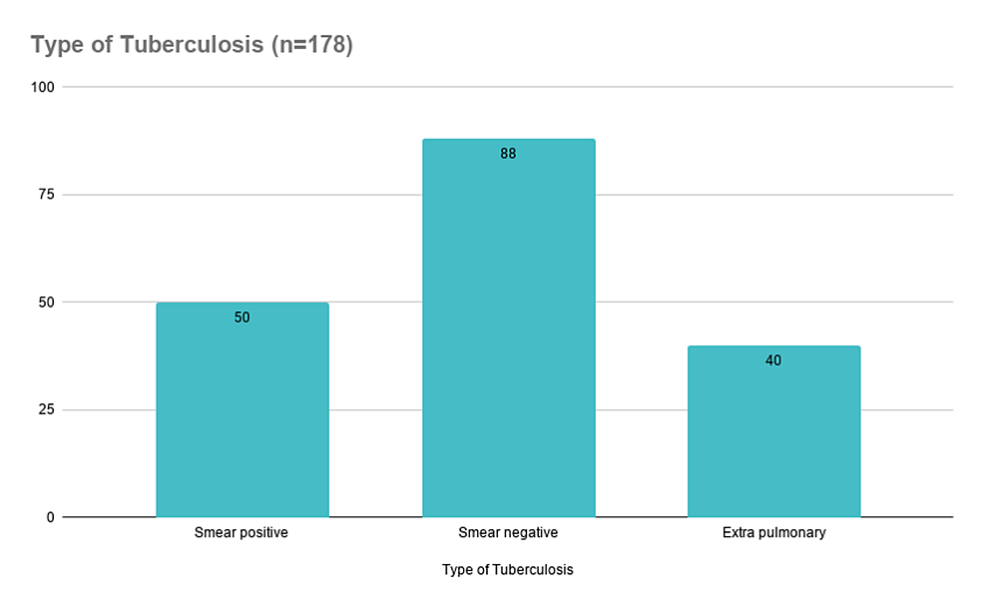
Type of tuberculosis infection among study participants

Fifty (50) patients had positive smears, 88 had smear-negative TB, while 40 patients had extrapulmonary tuberculosis (Figure 1).

We assessed the outcome and treatment response in TB patients with or without concomitant hepatitis B infection. It was found that patients with concomitant hepatitis B had significantly poorer outcomes as compared to patients who did not have hepatitis B (<0.001). Similarly, TB and hepatitis B patients were significantly associated with severe tuberculosis (<0.001) and required a higher frequency of retreatment (<0.001) (Table 2).

**Table 2:** The effect of hepatitis B viral infection on patients with tuberculosis

Variables	Hepatitis B Positive	Hepatitis B Negative	p-value
Treatment outcome			
Good	61 (68.54%)	79 (88.76%)	<0.001
Bad	28 (31.46%)	10 (11.24%)	
Severity of tuberculosis			
Severe	72 (80.90%)	19 (21.35%)	<0.001
Mild	17 (19.10%)	70 (78.65%)	
Retreatment category			
Yes	42 (47.19%)	6 (6.74%)	<0.001
No	47 (52.81%)	83 (93.26%)	

## Discussion

Patients suffering from pulmonary tuberculosis with an added hepatitis B infection are increasingly sensitive to the hepatotoxic effects of anti-tuberculous drugs. This is because hepatitis infection results in liver damage, making the organ more susceptible to drug-induced damage [10-12]. A study by Kim et al. reported that 13.7% of the patients under study who received anti-tuberculous drugs developed a drug-induced liver injury [13]. In patients with liver disease, tuberculosis treatment can increase the likelihood of liver failure. This would implicate the need for a delay in tuberculosis treatment in patients suffering from acute hepatitis [14]. However, a study has shown that patients who were treated with antiviral drugs soon after the diagnosis of tuberculosis had a lower risk of drug-induced liver injury caused by the hepatotoxic effects of anti-tuberculous drugs [15].

A study by Feleke et al. found that the prevalence of hepatitis B infection in patients with tuberculosis was 15.7%. The study noted that hepatitis B infection was commonly associated with alcohol abuse, sex, human immunodeficiency virus (HIV), and chronic illnesses [16]. The severity of tuberculosis infection in patients with hepatitis B was also evaluated by Feleke et al., It was found that tuberculosis infection when associated with hepatitis B had a severity of 80%. This finding was also supported by the study conducted by Al-Khazraji [17]. Both these results supported the conclusion of our study. The increase in the severity of infection implies that hepatitis infection may have the ability to reactivate and worsen the symptoms of tuberculosis, resulting in adverse clinical features and outcomes [18].

The study by Feleke also reported that the success of anti-tuberculous treatment in patients with a hepatitis B coinfection went down by 20.6% [16]. This study was also supported by research in China conducted by Chen et al., in the year 2018, which concluded that patients with a hepatitis B infection receiving anti-tuberculous treatment are prone to developing a loss of liver function and poor tuberculosis treatment outcomes [19]. These findings were consistent with our study, which indicated poor treatment outcomes in patients suffering from tuberculosis with an added hepatitis B infection, as compared to patients who were only suffering from tuberculosis.

The low success rate of tuberculosis treatment in patients with hepatitis B coinfection implies that these patients may have poor adherence to anti-tuberculous drugs, along with poor bioavailability and drug metabolism owing to the recurrent bouts of vomiting that commonly occur in hepatitis infections [20].

Our study reports a strong association between the treatment outcomes of patients with tuberculosis with an added hepatitis B infection. Additionally, the study highlighted the effect of hepatitis B virus infection on the prognosis of tuberculosis infection. There is a need for further research, which would identify the specific serotypes of hepatitis B infection in the patients of tuberculosis. The current study was limited due to the non-randomized selection of participants, hence, limiting the findings to the particular sample and making it not applicable to a larger population.

## Conclusions

Our study reports a strong association between the treatment response of patients with tuberculosis with an added hepatitis B infection. Additionally, the severity of the disease is also strongly impacted by the presence of hepatitis, which may impact the disease prognosis and outcome. Further multi, large-scale studies are required to explore the relationship between clinical and socio-demographic characteristics of patients and its impact on the outcome of TB/HBV patients.

## References

[1] Razavi-Shearer D, Gamkrelidze I, Nguyen MH (2018). Global prevalence, treatment, and prevention of hepatitis B virus infection in 2016: a modelling study. Lancet Gastroenterol Hepatol.

[2] (2021). WHO. Hepatitis B. http://who.int/news-room/fact-sheets/detail/hepatitis-b.

[3] Sulis G, Roggi A, Matteelli A, Raviglione MC (2014). Tuberculosis: epidemiology and control. Mediterr J Hematol Infect Dis.

[4] Kempker RR, Alghamdi WA, Al-Shaer MH, Burch G, Peloquin CA (2019). A pharmacology perspective on simultaneous tuberculosis and hepatitis C treatment. Antimicrob Agents Chemother.

[5] Zhang C, Li X, Liu Y, Qiao S, Chen Y, Zhou Y, Shen Z (2017). Co-infections of tuberculosis, hepatitis B or C viruses in a cohort of people living with HIV/AIDS in China: predictors and sequelae. AIDS Care.

[6] Ullah I, Javaid A, Tahir Z, Ullah O, Shah AA, Hasan F, Ayub N (2016). Pattern of drug resistance and risk factors associated with development of drug resistant Mycobacterium tuberculosis in Pakistan. PLoS One.

[7] Vermund SH, Altaf A, Samo RN, Khanani R, Baloch N, Qadeer E, Shah SA (2009). Tuberculosis in Pakistan: a decade of progress, a future of challenge. Sten H. Vermund.

[8] Mehmood S, Raza H, Abid F, Saeed N, Rehan HM, Javed S, Khan MS (2019). National prevalence rate of hepatitis B and C in Pakistan and its risk factors. J Public Health.

[9] Sharma SK, Balamurugan A, Saha PK, Pandey RM, Mehra NK (2002). Evaluation of clinical and immunogenetic risk factors for the development of hepatotoxicity during antituberculosis treatment. Am J Respir Crit Care Med.

[10] van Hest R, Baars H, Kik S (2004). Hepatotoxicity of rifampin-pyrazinamide and isoniazid preventive therapy and tuberculosis treatment. Clin Infect Dis.

[11] Streata I, Weiner J 3rd, Iannaconne M (2016). The CARD9 polymorphisms rs4077515, rs10870077 and rs10781499 are uncoupled from susceptibility to and severity of pulmonary tuberculosis. PLoS One.

[12] Kim WS, Lee SS, Lee CM (2016). Hepatitis C and not Hepatitis B virus is a risk factor for anti-tuberculosis drug induced liver injury. BMC Infect Dis.

[13] Pan L, Jia ZS, Chen L, Fu EQ, Li GY (2005). Effect of anti-tuberculosis therapy on liver function of pulmonary tuberculosis patients infected with hepatitis B virus. World J Gastroenterol.

[14] Saraceni C, Joshi TV, Spera MA, Hutchings J (20181). Mycobacterium tuberculosis and acute hepatitis b coinfection: challenges in treatment: 2191. Am J Gastroenterol.

[15] Lui GCY, Wong NS, Wong RYK (2020). Antiviral therapy for hepatitis B prevents liver injury in patients with tuberculosis and hepatitis B coinfection. Clin Infect Dis.

[16] Feleke BE, Feleke TE, Adane WG, Girma A (2020). Impacts of hepatitis B and hepatitis C co-infection with tuberculosis, a prospective cohort study. Virol J.

[17] Al-Khazraji A, Alkhawam H, Garrido B (2016). Hepatitis B virus reactivation in an inactive carrier of chronic HBV after the initiation of treatment for tuberculosis. J Investig Med.

[18] Pillai AA, Anania FA, Pearlman BL (2016). Caution: reactivation of hepatitis B during hepatitis C treatment with direct-acting antiviral therapy. Am J Gastroenterol.

[19] Chen L, Bao D, Gu L, Gu Y, Zhou L, Gao Z, Huang Y (2018). Co-infection with hepatitis B virus among tuberculosis patients is associated with poor outcomes during anti-tuberculosis treatment. BMC Infect Dis.

[20] Kirby BJ, Symonds WT, Kearney BP, Mathias AA (2015). Pharmacokinetic, pharmacodynamic, and drug-interaction profile of the hepatitis C virus NS5B polymerase inhibitor sofosbuvir. Clin Pharmacokinet.

